# A unique AI-based tool for automated segmentation of pulp cavity structures in maxillary premolars on CBCT

**DOI:** 10.1038/s41598-025-86203-8

**Published:** 2025-02-14

**Authors:** Airton Oliveira Santos-Junior, Rocharles Cavalcante Fontenele, Frederico Sampaio Neves, Saleem Ali, Reinhilde Jacobs, Mário Tanomaru-Filho

**Affiliations:** 1 Department of Restorative Dentistry, School of Dentistry, São Paulo State University (UNESP), Araraquara, São Paulo Brazil; 2 OMFS IMPATH Research Group, Department of Imaging and Pathology, Faculty of Medicine, University of Leuven, Leuven, Belgium; 3 Department of Propedeutics and Integrated Clinic, Division of Oral Radiology, School of Dentistry, Federal University of Bahia (UFBA), Salvador, Bahia Brazil; 4 Department of Restorative Dentistry, King Hussein Medical Center, Jordanian Royal Medical Services, Amman, Jordan; 5 Department of Oral and Maxillofacial Surgery, University Hospitals Leuven, Leuven, Belgium; 6 Department of Dental Medicine, Karolinska Institute, Alfred Nobels Allé 8, 141 04 Huddinge, Stockholm Sweden

**Keywords:** Artificial intelligence, Cone-beam computed tomography, Endodontics, Premolars, 3-D Imaging, Preclinical research, Cone-beam computed tomography

## Abstract

To develop and validate an artificial intelligence (AI)-driven tool for the automatic segmentation of pulp cavity structures in maxillary premolars teeth on cone-beam computed tomography (CBCT). One hundred and eleven CBCT scans were divided into training (n = 55), validation (n = 14), and testing (n = 42) sets, with manual segmentation serving as the ground truth. The AI tool automatically segmented the testing dataset, with errors corrected by an operator to create refined 3D (R-AI) models. The overall AI performance was assessed by comparing AI and R-AI models, and thirty percent of the test sample was manually segmented to compare AI and human performance. Time-efficiency of each method was recorded in seconds (s). Statistical analysis included independent and paired t-tests to evaluate the effect of tooth type on accuracy metrics and AI versus manual segmentation. One-way ANOVA with Tukey’s post hoc test was used for time efficiency analysis. A 5% significance level was used for all analyses.The AI tool demonstrated excellent performance with Dice similarity coefficients (DSC) ranging from 88% ± 7 to 93% ± 3 and 95% Hausdorff distances (HD) from 0.13 ± 0.06 to 0.16 ± 0.06 mm. Automated segmentation of maxillary second premolars performed slightly better than that of maxillary first premolars in terms of intersection over union (*p* = 0.005), DSC (*p* = 0.008), recall (*p* = 0.008), precision (*p* = 0.02), and 95% HD *(p* = 0.04). The AI-based approach showed higher recall *(p* = 0.04), accuracy *(p* = 0.01), and lower 95% HD than manual segmentation (*p* < 0.001). AI segmentation (42.8 ± 8.4 s) was 75 times faster than manual segmentation (3218.7 ± 692.2 s) (*p* < 0.001). The AI tool proved highly accurate and time-efficient, surpassing human expert performance.

## Introduction

The integration of a digital workflow in dentistry has revolutionized daily clinical practice, enhancing patient experiences^1–3^. Key advances in digital dentistry involve technologies, including intraoral scanners (IOS), cone-beam computed tomography (CBCT), three-dimensional (3D) impressions, augmented reality, and dynamic navigation systems^1,4,5^. In Endodontics, the adoption of a digital workflow has enabled more assertive diagnoses, the formulation of well-suited treatment plans with a notable reduction in working time, and heightened predictability in endodontic treatment outcomes^2,3,6^.

Among the steps in the digital workflow, the delineation (i.e., segmentation) of dentomaxillofacial hard-tissue structures in CBCT scans is acknowledged as a pivotal factor for generating highly accurate 3D models of the structures of interest. Conventionally, segmentation methods encompass semi- or fully-automated approaches to determine the correct thresholding of the object of study based on the grayscale values of voxels in CBCT scans^7–9^. However, the segmentation process is considered operator and CBCT device-dependent, often necessitating additional manual corrections that demand significant clinical time and are subject to human subjectivety^7–9^.

Technological advances in computational engineering have facilitated the development of artificial intelligence (AI)-driven tools based on convolutional neural network (CNN) models to overcome limitations inherent in traditional segmentation methods, simplifying the digital workflow^1,10,11^. CNN, as a special type of AI algorithm based on an artificial neural network, demonstrates considerable promise in automatically segmenting digital images^12^. Previous investigations reported the effective application of highly accurate and time-efficient CNN models in the automated segmentation of various anatomical and non-anatomical structures, including teeth^10,13–15^, dental implants^16^, alveolar bone^11^, mandibular canal^17,18^, maxillary sinus^19^, pharyngeal airway space^20^, and mandibular incisive canal^21^, on CBCT scans.

In this context, novel AI-driven tools offer a valuable alternative for automating the segmentation of pulp cavity structures (i.e., pulp chamber and root canal), significantly impacting clinical procedures and endodontic treatment outcomes. These tools are particularly valuable for accurately identifying the morphology of the root canal system on CBCT scans, including anatomically complex regions such as apical deltas, narrow canals and isthmuses^22^. Additionally, these tools might enhance pre-clinical training by providing students with more effective access to root canals. Additionally, they can assist in analyzing large datasets for studies on root canal anatomy in different populations, offering a more comprehensive understanding of endodontic anatomical complexities^7,23,24^. Maxillary premolars present considerable anatomical challenges during preparation, cleaning, and obturation due to their challenging root canal systems, which often include reduced apical diameter and root curvature^25,26^. Typically, these teeth have two roots, often with multiple canals, isthmuses, and accessory canals^27^. Canal morphology changes with age, with older patients showing increased calcification. Furthermore, males generally have a higher prevalence of multiple canals, highlighting gender-related morphological differences^27^. The complexity is further increased by the proximity of the maxillary first and second premolars to vital anatomical structures like the maxillary sinus^25^.

Generating highly accurate 3D models of pulp chambers and root canals in maxillary premolars can be a highly effective strategy in endodontic treatment, particularly for guided access, as it ensures precise localization of pulp cavity structures, minimizes unnecessary removal of hard dental tissue, and optimizes treatment outcomes. Additionally, these 3D models are invaluable for educational training, helping students better understand complex anatomical structures, and they enhance dentist-patient communication by allowing patients to visualize and comprehend proposed treatment plans more clearly. Despite the importance of accurate segmentation in digital endodontic workflows, there is still a significant shortage of clinically validated tools for automating this process, particularly in maxillary premolars. To address this gap, our study focuses on developing and validating an AI-driven tool specifically designed to improve reliability and efficiency in endodontic practice.

The primary objective of this investigation was to develop and validate a novel AI-based tool for the automatic segmentation of the pulp chamber and root canal in maxillary premolars on CBCT scans. The hypothesis of the study suggests that the novel AI-driven tool would offer pulp cavity segmentation for maxillary premolars with a performance below that of human experts in terms of accuracy and time efficiency.

## Materials and methods

### Ethical aspects

The research ethics committee of University Hospitals Leuven granted ethical approval for this investigation before its initiation (protocol number: S67798). The current study adheres to the ICH-GCP principles and the World Medical Association Declaration of Helsinki on medical research. Considering the anonymization of patient data conducted before any analysis, informed consent was not required.

### Dataset

**Fig. 1:**
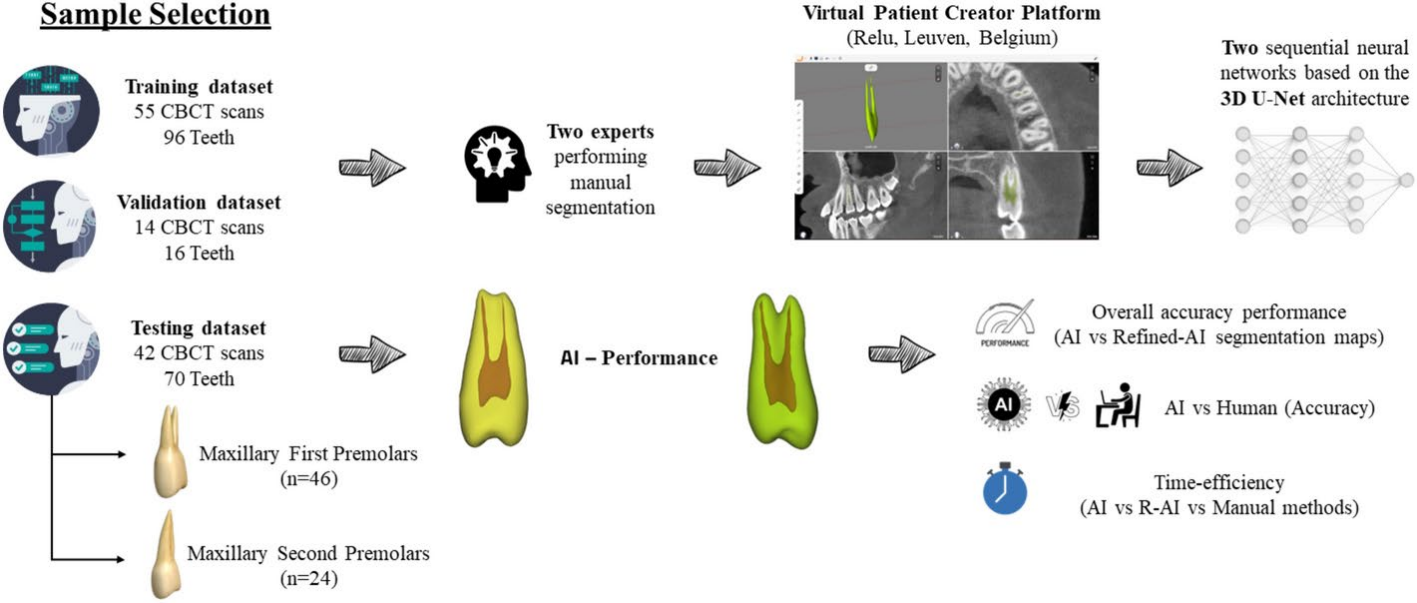
Conceptual framework of the study design for developing and validating an AI-driven tool for the automated segmentation of maxillary premolars.

Retrospectively, a set of 111 CBCT scans was retrieved from the Dentomaxillofacial Imaging Center database of UZ Leuven University Hospital in Leuven, Belgium. These scans were acquired for different purposes unrelated to the current investigation, such as endodontic treatment planning, implant planning, and oral and maxillofacial surgery procedures. The dataset was acquired using two CBCT devices: 3D Accuitomo 170 (J Morita, Kyoto, Japan) and NewTom VGi evo (Cefla, Imola, Italy). It is important to highlight that heterogeneous acquisition parameters were employed to obtain the CBCT scans: For 3D Accuitomo, 90 kilovoltage-peak (kVp), 5 milliampere (mA), field of view (FOV) of 8 × 8, 10 × 10, 14 × 10, and 17 × 12 cm, with a voxel size ranging from 0.125 to 0.250 mm. For the NewTom VGi EVO, the parameters comprised 110 kVp, 3 – 20 mA, FOV sizes of 8 × 8, 10 × 10, 12 × 8, 16 × 16, and 24 × 19 cm, with a voxel size ranging from 0.125 to 0.300 mm. Figure 1 summarizes the conceptual framework of the current study design.

For the selection of the imaging dataset, inclusion criteria encompassed CBCT scans from patients with a complete permanent dentition and satisfactory image quality, characterized by medium levels of sharpness and contrast, and low noise levels. This approach ensured accurate delineation of pulp chambers and root canals in maxillary premolars. CBCT scans with FOV covering either the maxilla alone or both the maxilla and mandible were included. Although some scans captured both arches, the analysis focused exclusively on maxillary premolars. Including scans with different FOVs aimed to enhance the generalizability of the study findings. Scans with poor image quality, such as those affected by significant artifacts from beam hardening or movement, were excluded.

The selected CBCT scans were exported in Digital Imaging and Communication in Medicine (DICOM) format and randomly distributed into the three steps of the CNN model:

- i) CNN Training (n = 55, 96 teeth): Training the AI model using manual segmentation carried out by operators as the ground truth.
- ii) CNN Validation (n = 14, 16 teeth): Conducting internal validation of the AI model by optimizing parameters until the establishment of an ideal architecture.
- iii) CNN Testing (n = 42, 70 teeth): Conducting the performance assessment of the AI model through the comparison of 3D models generated entirely by AI versus those obtained from refined automated segmentation (R-AI) performed by an expert.

The DICOM files were imported into the cloud-based online platform named as “Virtual Patient Creator” (Relu, Leuven, Belgium). This interactive platform offers a set of editing tools (e.g., brush, contour, and interpolation tools) for the manual segmentation of dentomaxillofacial anatomical structures on CBCT scans. Utilizing these tools enabled the precise delineation of the limits of the pulp chambers and root canals of maxillary premolars displayed in the multiplanar reconstructions of CBCT scans.

Each pulp chamber and root canal of the maxillary premolars teeth in the training and validation dataset of the CNN model underwent manual segmentation by two operators (F.S.N., and S.A.). Prior to performing manual segmentation on the ground truth sample, all operators underwent training and calibration using different CBCT scans not included in the study dataset. This calibration involved manual segmentation of 10 maxillary premolars on separate CBCT scans by the two operators (F.S.N. and S.A.) at two different time points. The segmentation results were compared to assess intra-examiner agreement (same operator at different times) and inter-operator agreement (between the two operators) using two metrics: intersection over union (IoU) and 95% Hausdorff distance (HD). Operators were considered calibrated if the IoU and 95% HD were at least 80% and 0.20 mm, respectively, for both intra- and inter-operator agreements. An oral radiologist with 8 years of experience (R.C.F.) reviewed all manual segmentations before CNN model development and made adjustments when deemed necessary. The final segmentation maps were exported in Standard Triangle Language (STL) format and later used as input for training the CNN model.

### CNN network architecture

The CNN model developed in this study was built based on two sequential neural networks based on the 3D U-Net architecture. Each network comprised four contracting encoder blocks and three expansive decoder blocks. These blocks included two convolutions with a standard kernel size (3 × 3 × 3), followed by rectified linear unit (ReLU) activation and group normalization with eight feature maps. The decision to adopt a two-step method stemmed from the challenges encountered when applying CNN to CBCT scans with a large FOV^10,11^.

The first neural network detected approximate pulp chambers and root canals, generating an initial segmentation model. Subsequently, the second neural network refined the initial segmentation, enabling automatic segmentation of the structures of interest at full resolution. The CNN models were implemented in PyTorch, and increased robustness of the AI algorithm was achieved using data augmentation strategies within the training dataset. These strategies included elastic deformation, rotation, scaling, cropping, and mirroring.

Moreover, the CNN model underwent optimization using the ADAM optimization algorithm. This process included reducing the learning rate and implementing early stopping based on the validation set to prevent overfitting and ensure the effective performance of the CNN model. Subsequently, the finalized CNN model was implemented and made accessible on the online cloud-based AI platform called "Virtual Patient Creator”.

### CNN model testing

Automated segmentation of pulp chambers and root canals of maxillary premolars was conducted using the aforementioned online platform. Each CBCT scan in DICOM format was uploaded to the platform, which then automatically segmented the pulp cavity structures for each maxillary premolar, generating individual 3D models in STL format. Additionally, the platform automatically recorded the time taken to generate the segmentation map in seconds.

An experienced oral radiologist with 8 years of experience (R.C.F.) evaluated the automated segmentations of the test set to detect and correct any errors, including oversegmentation or undersegmentation, in the AI-generated 3D models. After evaluating each automated segmentation, the operator determined that all segmentation maps required some form of minor correction.

For conducting this assessment, the resliceable axes tool within the "Virtual Patient Creator" platform was employed. By activating this tool, all CBCT multiplanar reconstructions (axial, sagittal, and coronal) were aligned to be parallel to the long axis each root canal. The brush tool was utilized to add or remove voxels in the segmentation maps, using the anatomical contour of the pulp cavity structures displayed on the CBCT reconstructions as reference. Finally, a new R-AI segmentation map of the pulp cavity structures of each maxillary premolar was obtained in STL format. A digital stopwatch was used to record the time taken on manual refinements.

### Validation metrics

A voxel-level confusion matrix was applied to evaluate the performance of the developed AI tool. The AI and R-AI 3D models were compared, and four variables were derived:

- False positive (FP): voxels initially identified as part of the pulp cavity structures by the CNN model but subsequently removed by the operator during the refinement of the AI segmentation.
- False negative (FN): Voxels not initially recognized as part of the pulp cavity structures by the CNN model but later included by the operator during the refinement of the AI segmentation.
- True positive (TP): Voxels representing the actual pulp cavity structures that were accurately segmented during the automated segmentation.
- True negative (TN): Voxels not associated with the pulp cavity structures and correctly excluded from the automated segmentation.

The performance of the developed CNN model was assessed using the following accuracy metrics based on the aforementioned variable values: IoU, Dice similarity coefficient (DSC), Recall, Precision, Accuracy, and 95% HD (Table 1).

**Table 1:** Accuracy metrics used to evaluate the performance of the AI-based tool.

Metrics	Definition	Formula
95% Hausdorff distance (HD)	The 95^th^ percentile of the maximum distance between a point on the AI segmentation map and its nearest point on the segmentation map is obtained after the expert performs automated segmentation refinement (R-AI). The strategy of using the 95^th^ percentile is to eliminate a possible subset of outliers. A 95% HD value of 0 mm means perfect segmentation	$\text{P}95(\text{min}\big\vert\mid p-g \mid\big\vert_2\,\text{u}\,\text{min}\big\vert \mid g-p \mid\big\vert_2)$ $g \epsilon G \:\:\:\:\:\: p \epsilon P$
Intersection over union (IoU)	The degree of overlap between the AI and R-AI segmentation maps. An IoU of 100% means perfect segmentation	$\text{IoU}= \frac{\text{TP}}{\text{TP}+\text{FP}+\text{FN}}$
Dice similarity coefficient (DSC)	The amount of intersection between the AI and R-AI segmentation maps. A DCS of 100% means a perfect intersection	$\text{DSC}=\frac{2\text{ x IoU}}{1+\text{ IoU}}$
Precision	The percentage of voxels correctly identified among all voxels considered to belong to the pulp chamber and root canal by the CNN model	$\text{Precision }= \frac{\text{TP}}{\text{TP }+\text{ FP}}$
Recall	The proportion of voxels belonging to the pulp chamber and root canal that were correctly identified by the CNN model	$\text{Recall }= \frac{\text{TP}}{\text{TP }+\text{ FN}}$
Accuracy	The proportion of voxels successfully recognized by the CNN model among all observed voxels	$\text{Accuracy }= \frac{\text{TP}+\text{TN}}{\text{TP }+\text{ TN}+\text{FP}+\text{FN}}$

### Comparison between human and AI-driven segmentations

The evaluation of AI-based automated segmentation performance involved a comparison with manual segmentation performed by a human (i.e., manual segmentation). Twenty-one teeth, constituting 30% of the test sample and including both maxillary first and second premolars, were randomly selected. An experienced endodontist with experience in CBCT image analysis (A.O.S.J.) manually performed the segmentation of pulp cavity structures using the aforementioned AI platform. The operator used the contour tool to manually outline the pulp chamber and root canal boundaries for each tooth based on the axial reconstructions of the CBCT scans.

Subsequently, the resliceable axes tool was employed to align each CBCT scan parallel to the long axis of each root canal. This allowed the operator to add or remove voxels while navigating the sagittal and coronal reconstructions of CBCT scans, facilitating the establishment of an ideal 3D model for each tooth. This task was performed twice, with a 30-day interval, to evaluate the accuracy of manual segmentation. The STL files obtained from the initial and subsequent segmentation sessions for each case were compared to calculate the accuracy metrics previously described. Finally, these manual segmentation results were compared with those obtained from automated segmentation by the CNN model for each accuracy metric. A digital stopwatch was used to record the time taken to manually segment the pulp chamber and root canal of each maxillary premolar tooth.

### Time-efficiency analysis

The comparison of the time needed for segmenting the pulp cavity structures of maxillary premolars was conducted for the different methods investigated: manual, AI, and R-AI methods. This analysis utilized the same sample (n = 21) employed for assessing the accuracy of manual and AI-driven segmentation:

- i) Manual segmentation: The time required for the operator to perform the manual segmentation of pulp cavity structures encompassed the duration from importing the DICOM data to the AI platform until obtaining the segmentation map.
- ii) AI segmentation: The online platform recorded the time spent for the automated segmentation of pulp cavity structures until obtaining the 3D model.
- iii) R-AI segmentation: The duration of manual refinements performed by the operator was recorded and combined with the time taken by the AI method.

### Statistical analysis

The analysis of data was conducted using SPSS statistical software (version 24.0, IBM Corp., Armonk, NY). Descriptive data analysis involved summarizing the results with mean and standard deviation (SD) values for accuracy and time-efficiency assessment.

The normal distribution of data was verified through the Shapiro–Wilk test. For the comparison of mean accuracy metric values between the maxillary first and second premolars, the independent t-test was utilized. Similarly, to compare the performance between AI-driven and manual approaches, the paired t-test was applied. Lastly, the one-way analysis of variance (ANOVA) with the Tukey post-hoc test was conducted to compare the time needed for pulp cavity structures segmentation among the segmentation methods investigated. A significance level of 5% was adopted for all analyses.

Using GPower statistical software (version 3.1.9.2, GPower, Düsseldorf, Germany), post hoc power analyses were performed for all statistical tests performed in the study as follows: for the independent t-test, the analysis considered the difference between group means, the SD, and the sample size for each group. For the paired t-test, the mean difference between paired observations, their SD, and the sample size for each accuracy metric were considered. For ANOVA, the power analysis considered the minimum difference between groups, the within-group SD, and the number of observations per group. Based on these parameters, the statistical power achieved ranged from 70 to 99%.

## Results

**Fig. 2:**
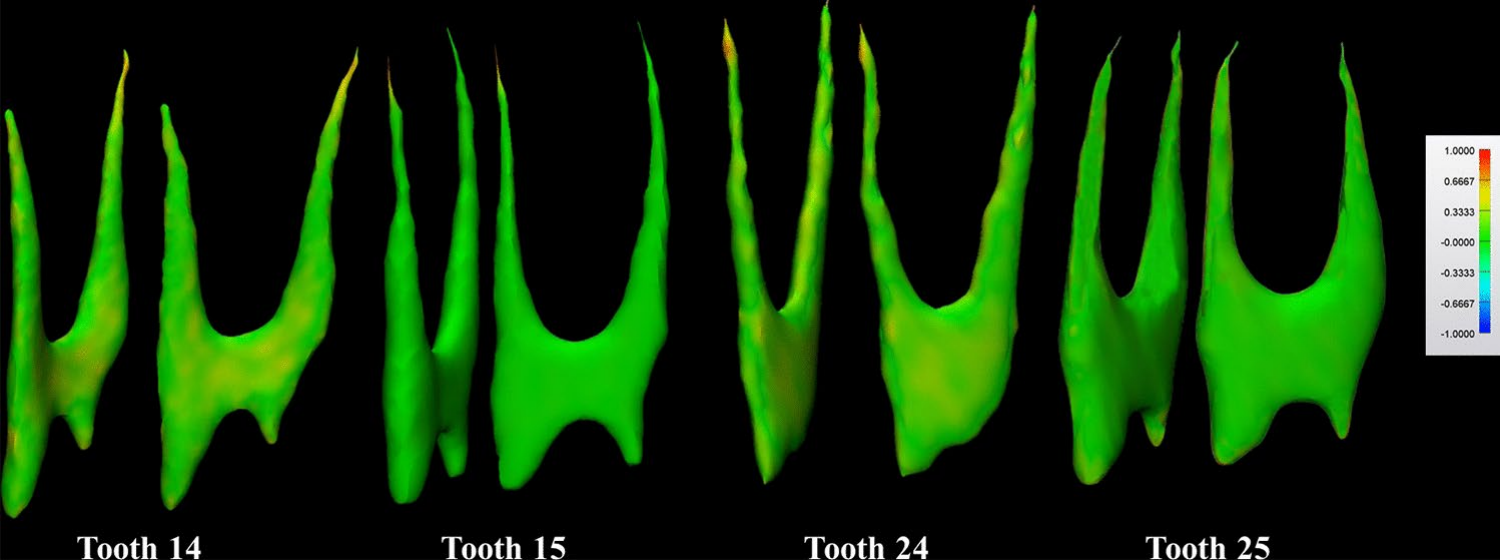
Comparison of STL files between AI and R-AI models for all types of maxillary premolars teeth groups (teeth 14, 15, 24, and 25) using color mapping in frontal and lateral views. Red and yellow areas indicate significant differences, highlighting discrepancies between the AI and R-AI 3D models.

Table 2 presents the performance of the AI-driven segmentation, showing the accuracy metrics for each maxillary premolar tooth group (i.e., first and second maxillary premolars). Regardless of the type of maxillary premolar and the accuracy metrics, the automated segmentation based on AI showed an excellent performance with high values of IoU (ranging from 80% ± 10 to 86% ± 5), DSC (ranging from 88% ± 7 to 93% ± 3), recall (ranging from 90% ± 8 to 95% ± 3), precision (87% ± 7 to 90% ± 4), and accuracy (99% ± 0.6 to 99% ± 1.0). Additionally, a low value of 95% HD was observed (ranging from 0.13 ± 0.06 mm to 0.16 ± 0.06 mm), confirming the similarity between AI and R-AI 3D models. This indicates a minor level of refinements needed in automated segmentation (Fig. 2).

**Table 2:** Evaluation of AI-driven automated maxillary premolar canal segmentation performance by teeth group using accuracy metrics.

Teeth group	IoU (%) * Mean (SD)	DSC (%) * Mean (SD)	Recall (%) * Mean (SD)	Precision (%) * Mean (SD)	Accuracy (%) Mean (SD)	95 HD (mm) * Mean (SD)
Maxillary First Premolar (n = 46, Teeth 14/24)	80 (10)	88 (7)	90 (8)	87 (7)	99 (1)	0.16 (0.06)
Maxillary Second Premolar (n = 24, Teeth 15/25)	86 (5)	93 (3)	95 (3)	90 (4)	99 (0.6)	0.13 (0.06)
*p*-value	0.005	0.008	0.008	0.02	0.87	0.04

IoU, Intersection over union; DSC, Dice similarity coefficient; HD, Hausdorff distance; n, sample size; SD, Standard deviation. Asterisk (*) indicates a statistically significant difference between the teeth groups (*p* < 0.05) within each accuracy metric. Statistical power analysis ranging from 70% (accuracy metric) to 98% (DSC metric).

**Fig. 3:**
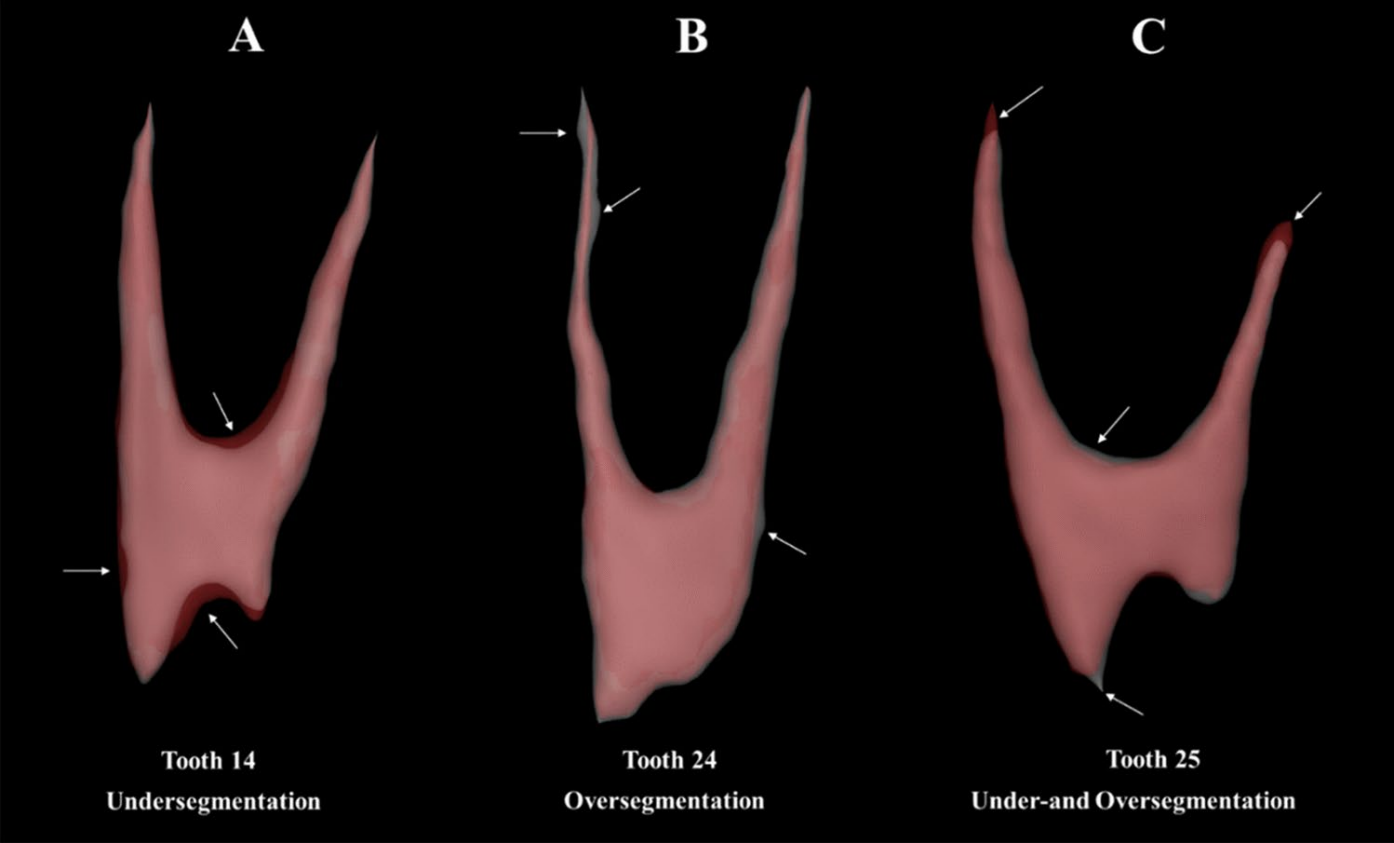
Three-dimensional models generated by AI illustrating errors in the automatic segmentation method. White areas (before refinements) are indicated by arrows alongside red areas (after refinements) in the root canal segmentation maps. A, Undersegmentation in the pulp chamber and root canal of the maxillary first right premolar tooth; B, Oversegmentation in the root canal of the maxillary first left premolar tooth; C, Both under- and oversegmentation in the pulp chamber and apical third of the maxillary second left premolar tooth.

Regarding the effect of the maxillary premolar tooth group, the maxillary first premolars (teeth 14 and 24) demonstrated inferior performance compared to the maxillary second premolars (teeth 15 and 25) regarding IoU (*p* = 0.005), DSC (*p* = 0.008), Recall (*p* = 0.02), and 95% HD (*p* < 0.001). The maxillary first premolars had lower values for IoU (80% ± 10), DSC (88% ± 7), recall (90% ± 8), and precision (87% ± 7) compared to the maxillary second premolars, which had higher values for IoU (86% ± 5), DSC (93% ± 3), recall (95% ± 3), and precision (90% ± 4). Additionally, the maxillary first premolars exhibited a higher 95% Hausdorff distance (HD) value (0.16 ± 0.06 mm) compared to the maxillary second premolars (0.13 ± 0.06 mm). In terms of the accuracy metric, there was no statistically significant difference observed among the groups of teeth (*p* = 0.87). An illustration of the types of errors found in the 3D models obtained by the AI-based method can be seen in Fig. 3.

**Fig. 4:**
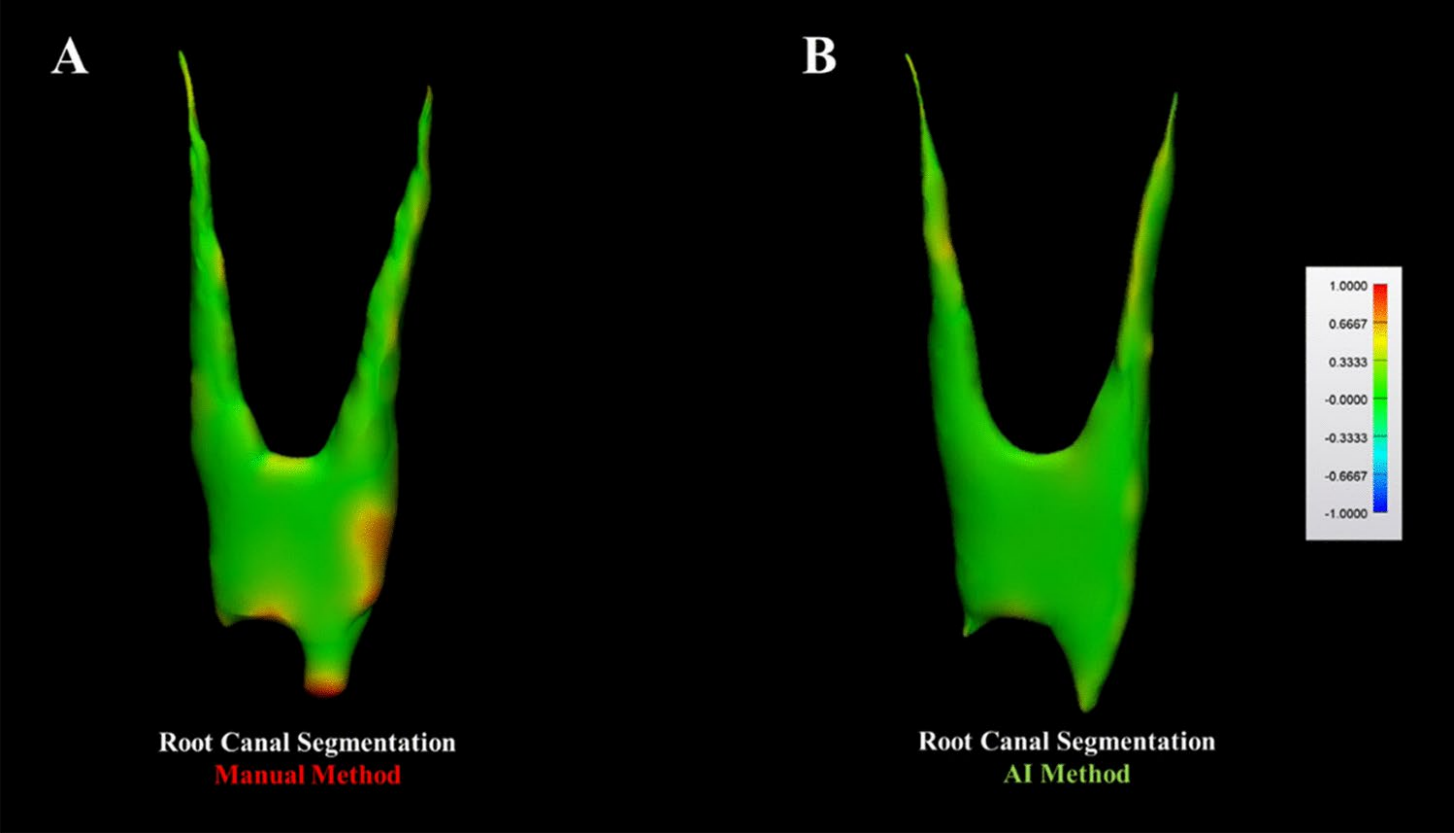
Comparison between manual (**A**) and AI (**B**) segmentation methods based on STL comparison using color mapping of a maxillary first right premolar tooth. The areas highlighted in red and yellow indicate significant differences between the first and second manual segmentation for the manual method and between the AI and R-AI 3D models for the AI method.

Table 3 presents the results comparing accuracy metrics between the manual and AI segmentation approaches. The AI method exhibited higher recall (91% ± 7, *p* = 0.04), accuracy (99% ± 1,* p* = 0.01), and lower 95% HD (0.14 ± 0.05 mm, *p* < 0.001) when compared to the manual approach (Fig. 4**).** However, there was no statistically significant difference observed between the segmentation methods tested in terms of IoU (*p* = 0.06), DSC (*p* = 0.09), and precision (*p* = 0.33) metrics.

**Table 3:** Comparison of manual and AI-driven segmentation methods for maxillary premolar canal segmentation, including all types of maxillary premolars (teeth 14, 15, 24, and 25).

Metrics	Manual Mean (SD) (n = 21)	AI Mean (SD) (n = 21)	*p*-value
IoU (%)	75 (7)	82 (0.9)	0.06
DSC (%)	86 (4)	90 (6)	0.09
Recall (%) *	86 (6)	91 (7)	0.04
Precision (%)	85 (7)	89 (6)	0.33
Accuracy (%) *	97 (1)	99 (1)	0.01
95% HD (mm) *	0.26 (0.06)	0.14 (0.05)	< 0.001

AI, Artificial intelligence; IoU, Intersection over union; DSC, Dice similarity coefficient; HD, Hausdorff distance; n, sample size; SD, Standard deviation. Asterisk (*) indicates a statistically significant difference between the segmentation methods within each accuracy metric (*p* < 0.05). Statistical power analysis of 99% for all metrics.

**Fig. 5:**
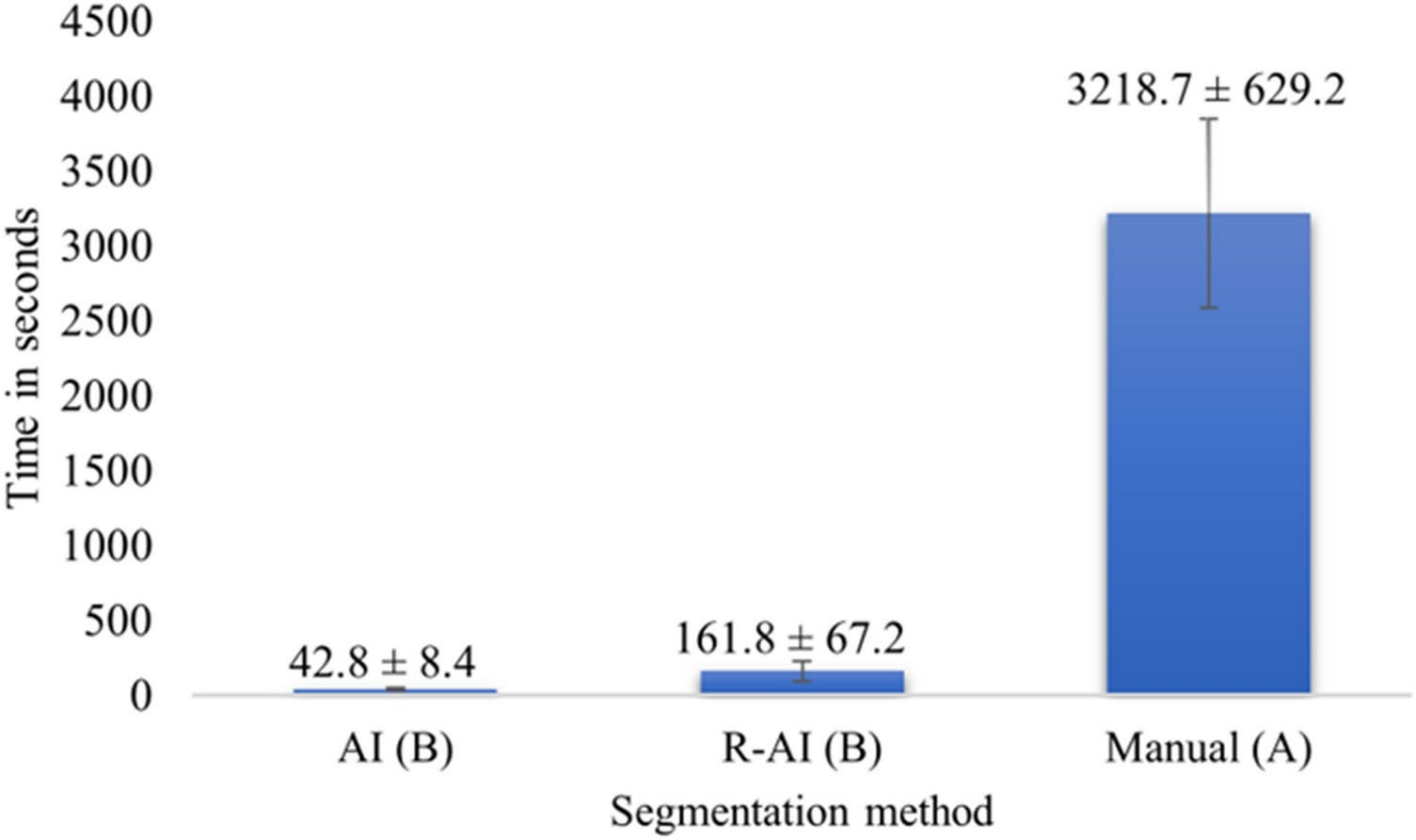
Time-efficiency analysis based on segmentation method. Different uppercase letters indicate statistically significant differences among segmentation methods (p < .05). Statistical power analysis of 0.99. AI, Artificial Intelligence; R-AI, Refined Artificial Intelligence.

Figure 5 depicts the time required for manual, AI, and R-AI segmentation methods. The time taken by AI-driven segmentation (42.8 ± 8.4 s) and R-AI (161.8 ± 67.2 s) showed similar working times (*p* = 0.95), both significantly shorter than the manual method (3218.7 ± 692.2 s), which proved to be the most time-consuming approach (*p* < 0.001).

## Discussion

Accurate segmentation of pulp cavity structures is a critical component in the digital workflow in Endodontics^7,23,24,28^. Obtaining precise 3D models of the pulp chamber and root canal of maxillary premolar teeth could significantly enhance diagnostic efficiency for clinicians, leading to higher success rates in endodontic treatment outcomes. However, the literature has highlighted notable limitations in 3D image segmentation using thresholding-based methods, such as semi-automatic or fully-automated methods^10,11,13^. Therefore, the present investigation developed and validated an AI-based tool for automated segmentation of the pulp cavity structures of maxillary premolar teeth on CBCT scans. The developed CNN model demonstrated outstanding performance, generating highly accurate 3D models of these fine endodontic structures in a time-efficient manner, surpassing human performance based on manual segmentation.

The innovative and promising results of this research can be attributed to the continuous curriculum learning of the developed AI algorithm^29^. Initially, the AI-based tool was trained for the automated segmentation of less complex root canals (i.e., teeth with single root canals). Gradually, the CNN model was consistently optimized until it could provide adequate segmentation of the root canals of maxillary premolar teeth, a task considered much more complex. Consequently, the current findings revealed that the developed AI tool was capable of providing highly accurate automated segmentation of the pulp cavity structures of maxillary premolars (IoU: ranging from 80% ± 10 to 86% ± 5; DSC: ranging from 88% ± 7 to 93% ± 3; and 95% HD: ranging from 0.13 ± 0.06 mm to 0.16 ± 0.06 mm).

Based on these findings, it is conceivable that the CNN model developed in this study could serve as a potent tool in clinical practice for the automatic segmentation of pulp chambers and root canals of maxillary first and second premolars, signifying a revolution in the digital workflow in Endodontics. The segmentation of endodontic structures on CBCT scans has consistently posed a challenge in the digital workflow^7,23,24,28^. A previous investigation proposed the use of two 3D U-Net networks for automated segmentation of the pulp chamber and root canal of single-rooted premolar teeth^7^. Although acceptable performance was reported (DSC = 87.49%, 95% HD = 1.99 mm), the authors employed 3D models obtained from micro-computed tomography data as the ground truth for evaluating the AI tool´s performance, limiting the generalizability of the results. Furthermore, the proposed CNN model struggled to provide accurate automated segmentation of the apical third of the root canals of single-rooted premolar teeth. Other studies assessed the performance of AI-based tools for automatic tooth segmentation^13^, single-rooted teeth and their root canals^23^, teeth and pulp chambers of single- and multi-rooted teeth^28^, and lower molars and their pulp chambers^30^ on CBCT scans. However, none of these aforementioned studies focused on the development and validation of an accurate and fast CNN model for automatic segmentation of the pulp chamber and root canal of maxillary premolar teeth on CBCT scans. Therefore, direct comparisons of the current findings with previous investigations were not feasible.

Overall, the accuracy metrics in this study indicated slightly superior performance of AI-based automated segmentation for maxillary second premolars compared to first premolars. However, it is crucial to highlight that this statistical difference was relatively low, mainly due to the low values of SD achieved and may not have significant influence on the performance of the CNN model developed in a clinical scenario. These results may be explained by the anatomical complexities of maxillary first premolars, such as the fact that most of the apical foramina do not coincide with the root apex, and also the significant incidence of apical delta canals^25,31^. Additionally, this group of teeth often presents anatomical challenges on the external surface of the root, including the presence of a pronounced depression on the palatal surface of the buccal root, which can have important clinical implications in endodontic procedures^25^. Thus, all these factors could contribute to the lower performance observed in the AI-driven segmentation of pulp cavity structures in maxillary first premolars.

Given the anatomical complexities of root canal systems, a thorough understanding of root canal morphology is crucial in digital endodontics, especially for maxillary premolars where such variations are common^25–27,31^. The advent of CBCT scans has significantly improved diagnostic accuracy by providing detailed 3D imaging that surpasses traditional methods^26,32^. Integrating CBCT scans into the management of challenging clinical cases not only enhances clinician confidence but also enhances success rates in endodontic treatment^32^. This technology allows for precise navigation of root canal intricacies, leading to better patient outcomes^26^. By offering a comprehensive view of anatomical complexities, CBCT scans support the careful selection of instrumentation techniques and optimize obturation processes, both essential for achieving favorable endodontic results^32^.

Manual segmentation performed by a human was used as a reference to evaluate the results of the automated segmentation provided by the AI tool developed in this study. Remarkably, the AI outperformed human intelligence, akin to a retake of the Turing test, where machines are compared to humans for conducting a specific task^33,34^. This superiority was evident through the high recall values (91% ± 7), accuracy (99% ± 1), and low 95% HD values (0.14  ± 0.05 mm). Such performance likely arises because human decision-making is inherently complex and can vary due to factors like experience level and emotional state^34^. These human elements can introduce inconsistencies, even among skilled professionals, while AI-driven tools provide consistent and precise outcomes without such variability. It is crucial to emphasize that accuracy metrics are essential for evaluating the performance of the developed CNN model. This study’s findings confirm that the AI tool is highly effective at accurately segmenting the pulp chamber and root canal in maxillary premolars, challenging the notion that human-based approaches are superior in this context. In terms of IoU, DSC, and precision metrics, no statistical differences were detected between the AI-driven segmentation and manual segmentation methods. However, even for metrics that did not exhibit a statistically significant difference, it is essential to note that the developed AI algorithm demonstrated excellent performance with significance values approaching the cut-off value determined at *p* < 0.05. Future investigations with a larger sample size are encouraged, as they could provide statistical confirmation of these results.

In this study, the AI algorithm was trained and validated using a dataset of 69 CBCT scans containing 112 teeth. To prevent overfitting, several strategies were employed, including ensuring dataset heterogeneity in terms of CBCT devices and acquisition parameters, partitioning the data for continuous monitoring, and using data augmentation techniques such as rotations, flips, and zooms. Early stopping was also applied as a regularization method to enhance training efficiency. A significant contributor to the results was the AI platform’s prior training on a larger dataset of 175 CBCT scans and 500 teeth, as detailed by Shaheen et al. (2021)^14^ and Fontenele et al. (2022)^10^. This earlier training equipped the AI with strong feature extraction capabilities and a deep understanding of tooth segmentation, which accelerated the learning process for endodontic structures. Consequently, the AI-driven tool was able to achieve saturation in the learning curve with a smaller sample size, ultimately optimizing the study’s outcomes.

Time efficiency is a crucial parameter for implementing new AI-driven tools in the digital workflow in Endodontics^13^. The present study was pioneering in evaluating and comparing the time required to segment the pulp chamber and root canal of maxillary premolars through various segmentation approaches. The analysis of time efficiency revealed a notable increase in speed for AI-driven segmentation (42.8 ± 8.4 s) in contrast to the manual method (3218.7 ± 692.2 s), indicating an impressive 75-fold reduction. The current findings also indicate that the R-AI segmentation method presented a low working time (161.8 ± 67.2 s), demonstrating the minor level of refinements needed. These results underscore the excellent performance of the AI algorithm developed for highly accurate automated segmentation of the endodontic structures investigated. Importantly, they suggested that when some fine-tuning of the segmentation map is necessary in clinical practice, it can be performed quickly and without consuming significant clinical time.

In the methodological design of this study, CBCT scans with varying FOV and voxel sizes were intentionally included to enhance the generalization of the developed AI tool. This approach aimed to ensure that the CNN model could perform well across different acquisition protocols commonly encountered in clinical practice. The dataset, sourced from two different CBCT devices, further contributed to the model’s generalization. However, it is important to acknowledge the limitations of this investigation. One significant limitation is the need for future research to optimize the AI algorithm for application across a broader range of CBCT devices, as its performance may vary depending on the specific device used. Additionally, the study did not address the potential impact of artifacts from high-density materials (e.g., orthodontic brackets, metallic crowns, dental implants, gutta-percha, and endodontic sealers with high radiopacity) on the accuracy and efficiency of the CNN model. Moreover, although the current study demonstrated excellent accuracy and time efficiency in segmenting maxillary premolars, these results should not be generalized to multi-rooted teeth (i.e., those with more than two canals). Therefore, future research should focus on developing CNN models specifically tailored to handle these more complex scenarios. The present study is based on the development and testing of an AI-driven tool, with an architecture based on multiple 3D U-Net models. Future studies could explore the performance of other AI techniques to potentially enhance the segmentation process. Finally, the scope of the study could be extended through multicenter studies, which are highly recommended. Such studies would allow for the collection of a larger and more diverse dataset, including populations with varying demographic characteristics. This broader data collection would significantly improve the generalization and robustness of the CNN model, ensuring its applicability in a wide range of clinical settings.

The clinical applicability of the pioneering results of the current investigation is related to the possibility of precise and fast localization of calcified root canals, preventing unnecessary removal of dental hard tissue, and potentially increasing the success rates of the guided endodontic access technique. Furthermore, clinicians could utilize highly accurate 3D models of pulp cavity structures for planning and monitoring endodontic treatment within routine clinical practice, providing patients with a greater understanding of endodontic therapy. It is crucial to underscore that while CBCT scan is not advisable for routine use in all endodontic cases, it becomes indispensable in instances of moderate to high complexity, where precise diagnosis and prognosis are critical, especially when periapical radiographs are insufficient for accurate diagnosis and treatment planning^32^. In these challenging scenarios, particularly when dealing with teeth that have complex root canal systems (e.g., maxillary premolars), the AI tool developed in this study provides significant value. By generating highly accurate 3D models, this technology serves as a crucial resource for clinicians, enhancing both treatment planning and execution. Additionally, the developed AI tool could serve as an invaluable resource in the educational field of endodontics. Students could use 3D models to optimize pre-clinical training on accessing root canals, thereby enhancing the teaching–learning process.

## Conclusions

The AI-driven tool developed and validated in this study achieved highly precise automatic segmentation of pulp cavity structures in maxillary premolars with minimal processing time. It demonstrated slightly better accuracy in segmenting maxillary second premolars compared to first premolars, with both results being clinically satisfactory. Notably, the AI outperformed human experts in manual segmentation, underscoring its potential to revolutionize endodontic procedures. This advancement is especially significant in clinical scenarios requiring minimally invasive techniques for precise root canal localization, particularly in cases of root canal obliteration where guided endodontic access is crucial.

## Data Availability

The datasets used and/or analysed during the current study available from the corresponding author on reasonable request.
